# Manual of Diseases of the Skin, from the French of M. M. Cazenave and Schedel

**Published:** 1845-12

**Authors:** 


### Bibliographical Notices.

*Manual of Diseases of the Skin,* from the French of M. M. Cazenave, and Schedel; with notes and additions by Thos. H. Burgess, M. D. Revised and corrected, with additional notes, by H. D. Bulkey, M. D. Lecturer on Diseases of the Skin, &c. New York: J. & H. G. Langly, 1846, pp. 341.

The work of which this is the third edition, is too well known to need commendation. The present volume is improved and rendered more desirable to the student and physician. Its size and cost make it suitable for a text book and manual upon this difficult and important part of medicine.

